# Supplementary material for: Anthocyanins Profiling Analysis and RNA-Seq Revealed the Dominating Pigments and Coloring Mechanism in Cyclamen Flowers
Source: Biology (Basel). 2022 Nov 28;11(12):1721. doi: 10.3390/biology11121721 (PMC9774537; doi:10.3390/biology11121721)
Supplement: Supplementary file 1 [file biology-11-01721-s001.zip › biology-2023840-supplementary.pdf]

Supplementary Material

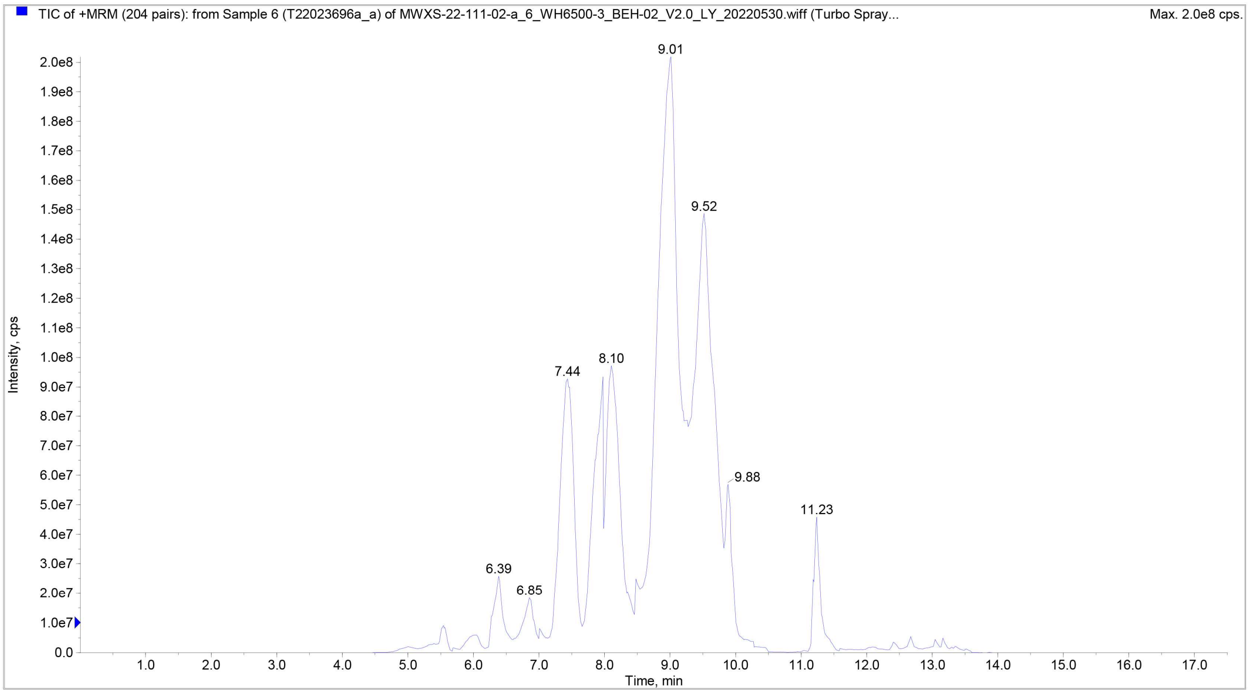

Figure S1: HPLC chromatogram of anthocyanin identified from C. purpurascens flower (BXK1).

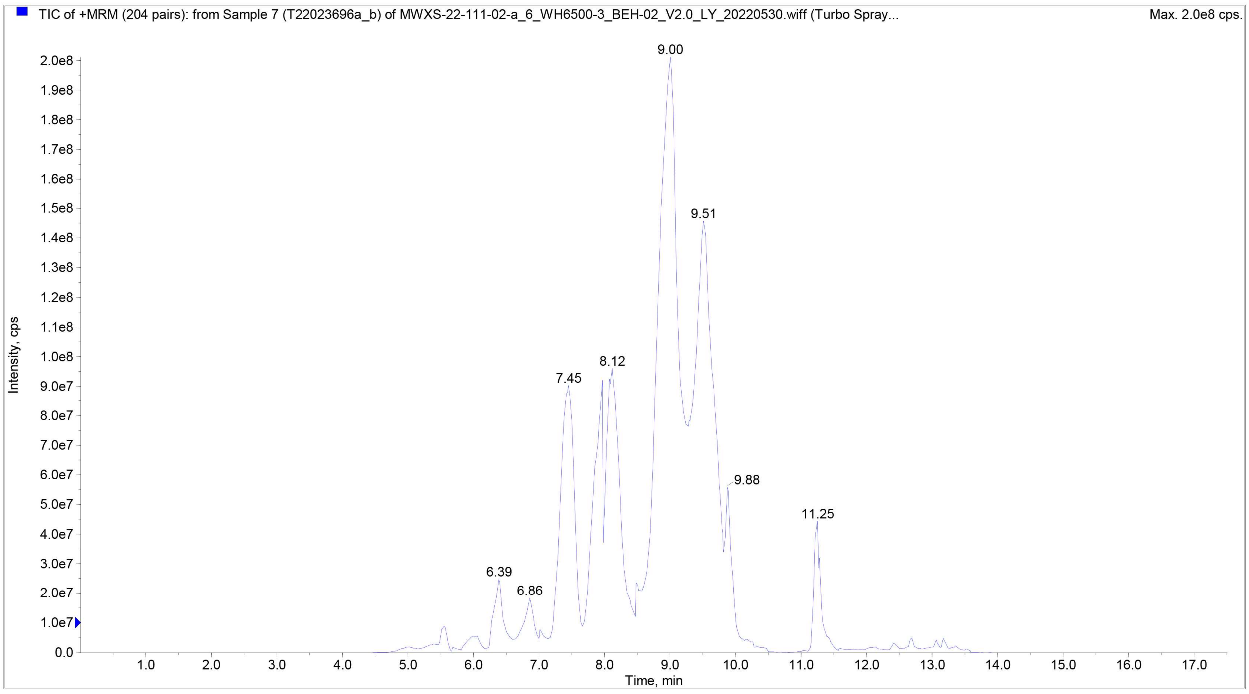

Figure S2: HPLC chromatogram of anthocyanin identified from C. purpurascens flower (BXK2).

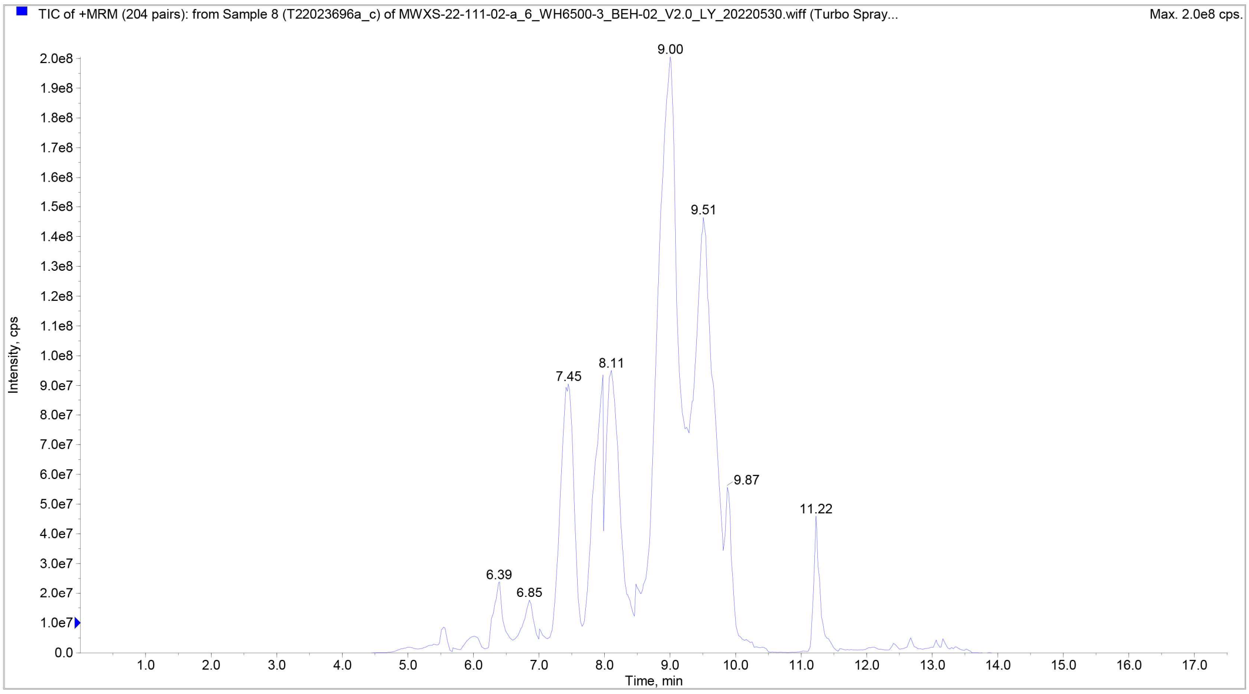

Figure S3: HPLC chromatogram of anthocyanin identified from C. purpurascens flower (BXK3).

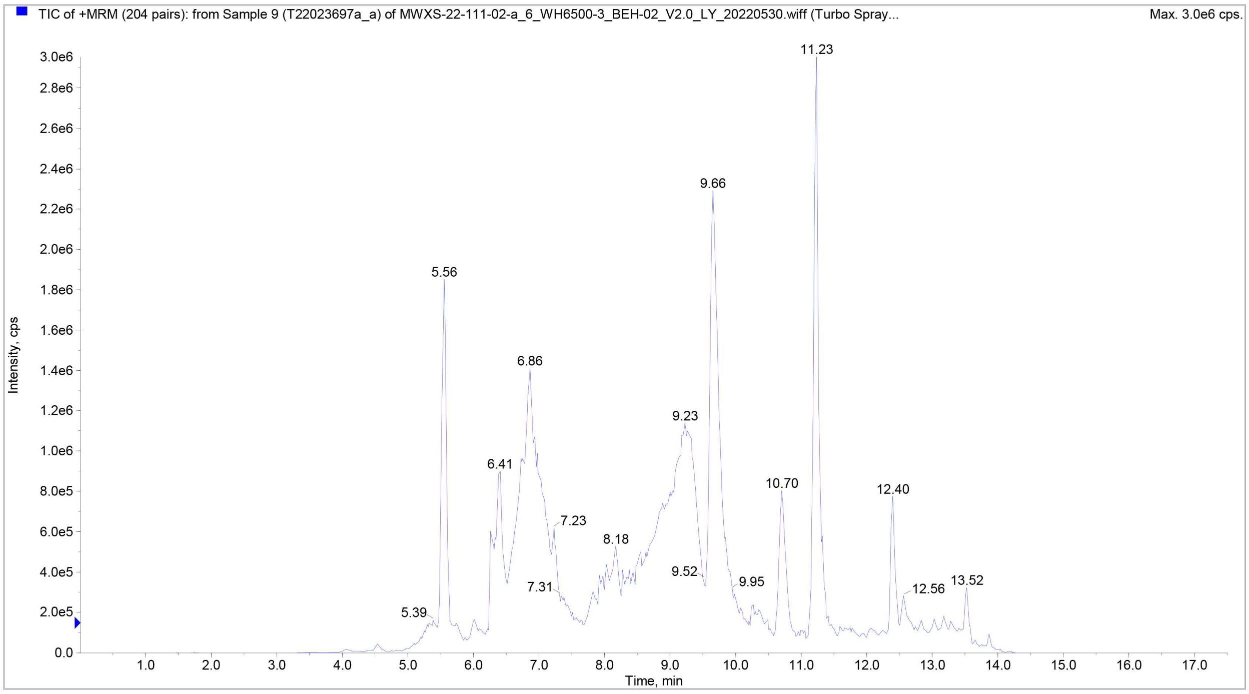

Figure S4: HPLC chromatogram of anthocyanin identified from C. purpurascens flower (HXK1).

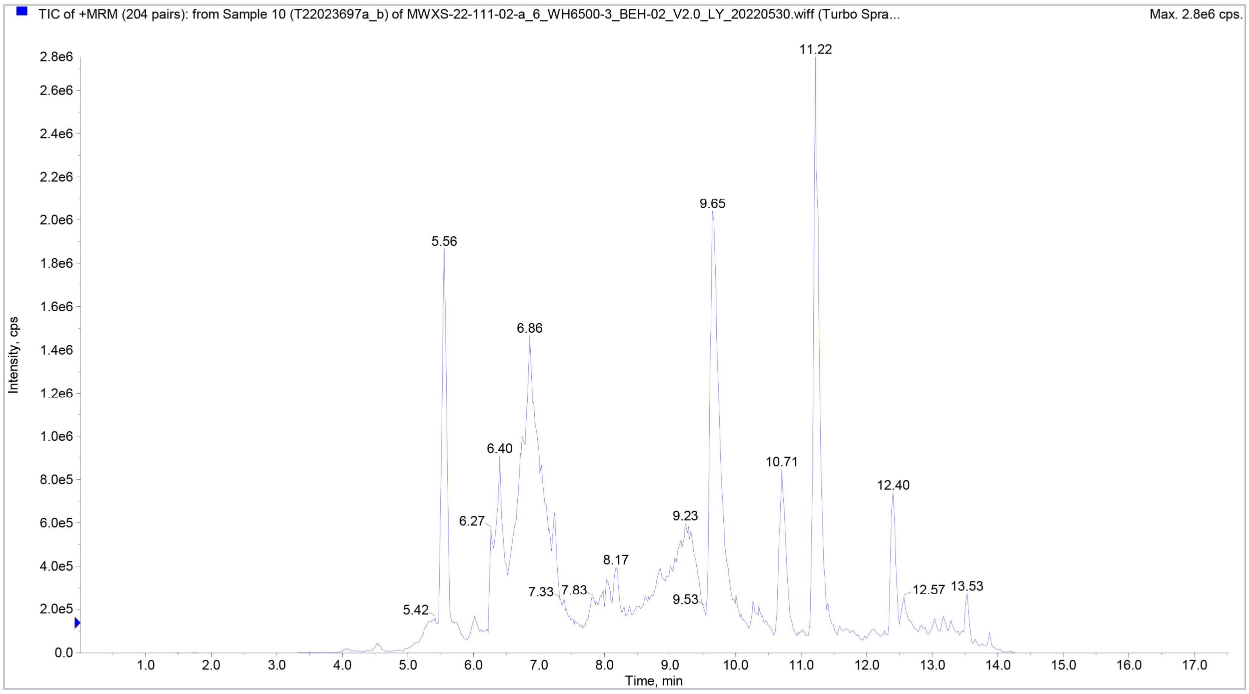

Figure S5: HPLC chromatogram of anthocyanin identified from C. purpurascens flower (HXK2).

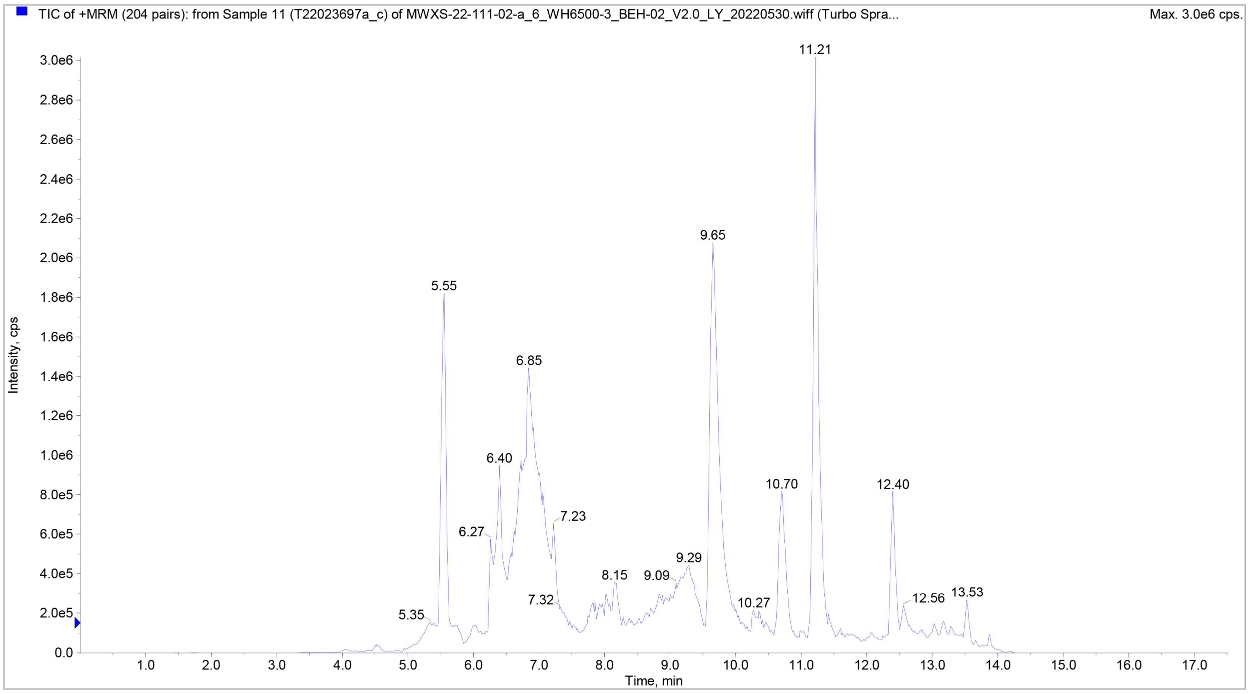

Figure S6: HPLC chromatogram of anthocyanin identified from C. purpurascens flower (HXK3).

Supplementary Table S1. Primers used in quantitative RT-PCR analysis

Gene ID	Sequence of 5' primer	Sequence of 3' primer
g1022_i0	TGGGGAAATTCATCCACATT	CACATTTTGGCTTCATGTGC
g10365_i0	GGCAATTCATGTTGCCTCTT	CGGGAGGTACAGTCAGGTGT
g10266_i0	CCTGCTGCTGAATGATTTTG	ATTACCCATCCCACCACAAC
g10340_i0	GAATGCAGGGCCATTGTACT	TGGTCCGTCTCCCAGAATAC
g10589_i0	TGATCGAGACCCGAAAAATC	TCAGCCGTCCATTCTTCTCT
g11358_i0	ACCCGAAAAGCAGAGTAGCA	TGCTGTCTTGTCCTCGTTTG
g34436_i0	AGCTGTTGTCGTTCATGCTG	TTTTGTCTCGAACCCAGACC
g18620_i0	CTCCGTGGTGGATTCTGATT	TTGGGCAAACCAACACATAA
g526_i0	GGTTAACTCGGGACAAAGCA	ACATTGCCACATCAACCTCA
eEF1α	CTGGTGGTTTTGAGGCTGG	CTGGCCAGGGTGGTTCATGAT
g5626_i0	GGTCGGAGTGTTTGAGCAAT	ATGGACGCTTGAACAAAACC
g8206_i0	GGTCGGAGTGTTTGAGCAAT	ATGGACGCTTGAACAAAACC

Supplementary Table S2.The candidate TFs involved in anthocyanin accumulation.

Transcription factor	Gene ID	Annotation	Up or Down-regulated TFs in BXK-VS-HXK
SRF	g31608_i0	MADS-domain transcription factor	up
	g27602_i0	MADS-domain transcription factor	up
	g23754_i0	like MADS-box protein AGL8	up
	g19111_i0	like MADS-box protein AGL15	up
	g26811_i0	like MADS-box protein AGL12	up
	g28187_i0	MADS-domain transcription factor	up

	g20026_i0	like MADS-box protein AGL66	up
MYB	g25403_i0	flavonoid-related R2R3 MYB 4a repressor transcription factor	up
	g6939_i0	Myb-related protein 3R-3	up
	g21772_i0	Myb-related protein 306	up
	g10657_i0	Myb-related protein 3R-3	up
	g27153_i0	Myb-related protein 73	up
	g6484_i0	Myb-related protein 3R-5	up
	g20876_i0	Myb-related protein 102	up
	g32204_i0	Myb-related protein 77	up
	g29503_i0	Atypical R2R3-MYB transcription factor CDC5	up
	g25712_i0	Myb-related protein 308	up
	g30397_i0	Myb-related protein 73	up
	g18595_i0	Myb-related protein MYB3R-2	up
	g18251_i0	Myb-related protein 41	up
	g21878_i0	Myb-related protein 3R-3	up
MBD	g15098_i0	Histone H3-K9 methyltransferase 4	up
	g7947_i0	C2H2 zinc finger-SET histone methyltransferase	up
Homeobox	g7677_i0	like homeodomain protein	up
	g25065_i0	homeobox-leucine zipper protein HAT5-like	up
	g11677_i0	like homeodomain protein	up
	g7543_i0	like homeodomain protein	up
	g14735_i0	Homeobox protein knotted-1-like 3	up
	g21714_i0	Homeobox protein HOS16	up

	g26153_i0	Homeodomain transcription factor ATHB-13	up
	g12891_i0	like homeodomain protein	up
CSD	g23776_i0	Cold shock domain-containing protein 3	up
TF_others	g33674_i0	Thymocyte nuclear protein 1	up
	g31650_i0	Protein MHF1 homolog	up
	g42606_i0	Histone H3.1	up
SRF	g33460_i0	like MADS-box protein AGL2	down
	g26358_i0	MADS-box transcription factor	down
C2H2	g2452_i0	Probable protein arginine N-methyltransferase 3	down
MYB	g2206_i0	transcription factor R2R3-MYB1	down
	g33866_i0	MYB family transcription factor	down
	g8716_i0	transcription factor R2R3-MYB1	down
	g5782_i0	MYB family transcription factor	down
	g27919_i0	Myb-related protein 66	down
	g19104_i0	Myb-related protein 61	down
	g34207_i0	Myb-related protein 23	down
	g30143_i0	MYB-like protein	down
	g17765_i0	MYB-like protein	down
	g36054_i0	transcription factor R2R3-MYB1	down
MBD	g7186_i0	Histone-lysine N-methyltransferase SUVR4	down
	g5449_i0	Histone-lysine N-methyltransferase family member protein	down
	g8491_i0	Histone H3-K9 methyltransferase 4	down
HSF	g11233_i0	Heat stress transcription factor B-2a	down

	g2691_i0	hypothetical protein TanjilG_28154	down
	g13489_i0	Heat shock transcription factor 30	down
	g11233_i0	Heat stress transcription factor B-2a	down
Homeobox	g22517_i0	Homeobox protein like	down
	g11440_i0	BEL1-like homeodomain protein 2	down
HMG	g5303_i0	WD repeat and HMG-box DNA-binding protein like	down
	g31522_i0	HMG1/2-like protein	down
	g5082_i0	Histone-lysine N-methyltransferase	down
E2F	g14734_i0	E2F transcription factor-3v	down
	g16173_i0	Transcription factor E2FC	down
TF_others	g20707_i0	Prohibitin-1 like	down
	g24930_i0	NAC transcription factor 037	down
	g19760_i0	ethylene-responsive transcription factor ERF014-like	down
	g36811_i0	transcription factor TCP4-like	down
	g32312_i0	ethylene-responsive transcription factor ERF014-like	down
	g2629_i0	Chloroplastic small ribosomal subunit protein cS22	down
	g22909_i0	Bromodomain and PHD finger-containing protein 1	down

Supplementary Table S3. The classification and quantification results of all detected metabolites.

Compounds	HXK_1	HXK_2	HXK_3	BXK_1	BXK_2	BXK_3	MWXS2211102a_R1	MWXS2211102a_R2	MWXS2211102a_R3
Cyanidin-3,5-O-diglucoside	6.67865014	6.70177614	6.17673349	0.790998071	0.954378405	0.864535388	1.643362	1.987148	2.0508
Cyanidin-3-O-galactoside	N/A	N/A	N/A	N/A	N/A	N/A	N/A	N/A	N/A
Cyanidin-3-O-arabinoside	N/A	N/A	N/A	N/A	N/A	N/A	N/A	N/A	N/A
Cyanidin-3-O-(6-O-p-coumaroyl)-glucoside	N/A	N/A	N/A	N/A	N/A	N/A	N/A	N/A	N/A
Cyanidin-3-O-5-O-(6-O-coumaroyl)-diglucoside	N/A	N/A	N/A	N/A	N/A	N/A	N/A	N/A	N/A
Cyanidin-3,5,3'-O-triglucoside	N/A	N/A	N/A	N/A	N/A	N/A	N/A	N/A	N/A
Cyanidin-3-(6"-caffeylsophoroside)-5-glucoside	N/A	N/A	N/A	N/A	N/A	N/A	N/A	N/A	N/A
Cyanidin-3-(6-O-p-caffeoyl)-glucoside	N/A	N/A	N/A	N/A	N/A	N/A	N/A	N/A	N/A
Cyanidin-3-O-sambubioside-5-O-glucoside	0.209188361	0.207948727	0.188731844	N/A	N/A	N/A	0.0751756	0.0774764	0.0846462
Cyanidin-3-O-xyloside	0.0497923554	0.041905496	0.0478527769	N/A	N/A	N/A	0.01932018	0.01820286	0.01874022
Cyanidin-3-O-sambubioside	31.1043733	29.6426273	29.3184358	N/A	N/A	N/A	9.69658	9.61404	9.64202
Cyanidin-3-O-rutinoside-5-O-glucoside	0.590881543	0.48689008	0.487755504	N/A	N/A	N/A	0.167724	0.1750132	0.1960626
Cyanidin-3-O-rutinoside	188.051653	188.794236	169.853434	N/A	N/A	N/A	74.7308	77.1604	75.5484
Cyanidin-3-O-(6-O-malonyl-beta-D-glucoside)	1.25754132	1.16968834	1.14177456	N/A	N/A	N/A	0.335902	0.3447	0.349088
Cyanidin-3-O-sophoroside	25.7269972	24.1956434	23.4085771	N/A	N/A	N/A	7.91688	7.82374	7.96414
Cyanidin-3-O-glucoside	117.53719	112.72118	109.879724	N/A	N/A	N/A	39.982	41.069	39.7926
Cyanidin-3-O-(6"-ferulylsophoroside)-5-glucoside	N/A	N/A	N/A	N/A	N/A	N/A	N/A	N/A	N/A
Delphinidin-3-O-(6-O-acetyl)-glucoside	N/A	N/A	N/A	N/A	N/A	N/A	N/A	N/A	N/A
Delphinidin-3-O-(6-O-p-coumaroyl)-glucoside	N/A	N/A	N/A	0.0379070878	0.0410557207	0.0355195474	N/A	N/A	N/A
Delphinidin-3-O-glucoside	22.234022	20.420811	19.974466	0.125856798	0.121228826	0.113361579	6.6097	6.79302	7.01426
Delphinidin-3-O-galactoside	0.226448003	0.246798257	0.243819257	0.0245004822	0.0266362556	0.027047376	0.0610562	0.07269	0.0681126
Delphinidin-3-O-sambubioside	0.0994400826	0.0897302279	0.0867180414	N/A	N/A	N/A	0.0272918	0.0317702	0.0314032
Delphinidin-3-O-rutinoside	0.131274793	0.129386729	0.134138679	N/A	N/A	N/A	0.0445756	0.040671	0.0455502

Delphinidin-3-O-sophoroside	0.232196625	0.255207775	0.240538285	0.0311608968	0.0292425458	0.0287276842	0.0623244	0.0649376	0.0755346
Delphinidin-3,5-O-diglucoside	N/A	N/A	N/A	0.0651065574	0.0619846459	0.0580760713	N/A	N/A	N/A
Delphinidin-3-O-(6-O-malonyl-beta-D-glucoside)	N/A	N/A	N/A	N/A	N/A	N/A	N/A	N/A	N/A
Delphinidin-3-O-rutinoside-5-O-glucoside	N/A	N/A	N/A	N/A	N/A	N/A	N/A	N/A	N/A
Delphinidin-3-O-rhamnoside	N/A	N/A	N/A	N/A	N/A	N/A	N/A	N/A	N/A
Delphinidin	0.719614325	0.665613271	0.684380546	N/A	N/A	N/A	0.220138	0.21131	0.1888878
Delphinidin-3-O-arabinoside	N/A	N/A	N/A	N/A	N/A	N/A	N/A	N/A	N/A
Delphinidin-3-O-5-O-(6-O-coumaroyl)-diglucoside	N/A	N/A	N/A	N/A	N/A	N/A	N/A	N/A	N/A
Delphinidin-3-O-(6-O-malonyl)-glucoside-3'-glucoside	N/A	N/A	N/A	N/A	N/A	N/A	N/A	N/A	N/A
Delphinidin-3-O-sambubioside-5-O-glucoside	N/A	N/A	N/A	N/A	N/A	N/A	N/A	N/A	N/A
Malvidin	N/A	N/A	N/A	N/A	N/A	N/A	N/A	N/A	N/A
Malvidin-3-O-(6''-acetylglucoside)-5-glucoside	N/A	N/A	N/A	N/A	N/A	N/A	N/A	N/A	N/A
Malvidin-3-O-(6-O-malonyl-beta-D-glucoside)	53.9538567	50.7831769	49.9648373	0.169021697	0.165303616	0.169188734	15.2102	15.4685	15.67814
Malvidin-3-O-rutinoside	N/A	N/A	N/A	N/A	N/A	N/A	N/A	N/A	N/A
Malvidin-3-O-sambubioside	N/A	N/A	N/A	N/A	N/A	N/A	N/A	N/A	N/A
Malvidin-3-O-sophoroside	N/A	N/A	N/A	N/A	N/A	N/A	N/A	N/A	N/A
Malvidin-3-O-galactoside	68.5210055	63.6357239	65.1189616	N/A	N/A	N/A	N/A	N/A	N/A
Malvidin-3-O-5-O-(6-O-coumaroyl)-diglucoside	N/A	N/A	N/A	N/A	N/A	N/A	N/A	N/A	N/A
Malvidin-3-O-sambubioside-5-O-glucoside	N/A	N/A	N/A	N/A	N/A	N/A	N/A	N/A	N/A
Malvidin-3,5-O-diglucoside	33.9221074	31.8614611	32.3312521	16.164947	17.9812779	17.3049109	12.23248	12.71612	13.61676
Malvidin-3-O-(6-O-p-coumaroyl)-glucoside	0.308038567	0.301802949	0.292189287	N/A	N/A	N/A	0.1001608	0.0948152	0.1052462
Malvidin-3-O-glucoside	2969.14256	2746.50134	2602.55997	N/A	N/A	N/A	912.488	911.204	932.678
Malvidin-3-O-arabinoside	0.329269284	0.293451072	0.30815511	N/A	N/A	N/A	0.083391	0.0841298	0.0958266
Pelargonidin-3-O-(6''-ferulylsambubioside)-5-O-(malonyl)-glucoside	N/A	N/A	N/A	N/A	N/A	N/A	N/A	N/A	N/A
Pelargonidin-3-O-sambubioside-5-O-glucoside	N/A	N/A	N/A	N/A	N/A	N/A	N/A	N/A	N/A
Pelargonidin-3-O-sophoroside-5-O-(malonyl)-glucoside	N/A	N/A	N/A	N/A	N/A	N/A	N/A	N/A	N/A
Pelargonidin-3-O-rutinoside-5-O-glucoside	N/A	N/A	N/A	N/A	N/A	N/A	N/A	N/A	N/A

Pelargonidin-3-O-sophoroside	N/A	N/A	N/A	N/A	N/A	N/A	N/A	N/A	N/A
Pelargonidin-3-O-[6-O-feruloyl-2-O-glucosyl-glucoside]-5-O-glucoside	N/A	N/A	N/A	N/A	N/A	N/A	N/A	N/A	N/A
Pelargonidin-3-O-(6-O-malonyl-beta-D-glucoside)	N/A	N/A	N/A	N/A	N/A	N/A	N/A	N/A	N/A
Pelargonidin-3-(6''-caffeylsophoroside)-5-glucoside	N/A	N/A	N/A	N/A	N/A	N/A	N/A	N/A	N/A
Pelargonidin-3-sophoroside-5-glucoside	N/A	N/A	N/A	N/A	N/A	N/A	N/A	N/A	N/A
Pelargonidin-3-O-5-O-(6-O-coumaroyl)-diglucoside	N/A	N/A	N/A	N/A	N/A	N/A	N/A	N/A	N/A
Pelargonidin-3-O-(6-O-p-coumaroyl)-glucoside	N/A	N/A	N/A	0.0856152363	0.0898553739	0.0974833895	0.01460016	0.01454144	0.0188695
Pelargonidin-3-O-galactoside	N/A	N/A	N/A	0.0713129219	0.0740544824	0.070238806	N/A	N/A	N/A
Pelargonidin	0.0690530303	0.0608977882	0.0590463359	N/A	N/A	N/A	N/A	N/A	N/A
Pelargonidin-3-O-glucoside	0.0616456612	0.063786193	0.0663115347	N/A	N/A	N/A	0.0236444	0.01987832	0.0224042
Pelargonidin-3-O-rutinoside	0.46255854	0.46916555	0.536845219	N/A	N/A	N/A	0.1680384	0.1833262	0.182968
Pelargonidin-3-O-sambubioside	3.49349174	3.25599531	3.265442	N/A	N/A	N/A	0.999726	1.036088	1.00904
Pelargonidin-3-O-[2-O-glucosyl-6-O-p-coumaroyl-glucoside]-5-O-glucoside	N/A	N/A	N/A	N/A	N/A	N/A	N/A	N/A	N/A
Pelargonidin-3-O-arabinoside	N/A	N/A	N/A	N/A	N/A	N/A	N/A	N/A	N/A
Pelargonidin-3,5-O-diglucoside	0.0240121901	0.0228171247	0.0223257969	0.573437801	0.634190193	0.584597978	0.1197752	0.1395326	0.1194548
Peonidin	N/A	N/A	N/A	N/A	N/A	N/A	N/A	N/A	N/A
Peonidin-3-O-galactoside	N/A	N/A	N/A	N/A	N/A	N/A	N/A	N/A	N/A
Peonidin-3-O-sambubioside	N/A	N/A	N/A	N/A	N/A	N/A	N/A	N/A	N/A
Peonidin-3-O-sophoroside	N/A	N/A	N/A	N/A	N/A	N/A	N/A	N/A	N/A
Peonidin-3-O-sambubioside-5-O-glucoside	N/A	N/A	N/A	N/A	N/A	N/A	N/A	N/A	N/A
Peonidin-3-O-(6''-ferulylsophoroside)-5-glucoside	N/A	N/A	N/A	N/A	N/A	N/A	N/A	N/A	N/A
Peonidin-3-O-P-hydroxybenzoylsophoroside-5-glucoside	N/A	N/A	N/A	N/A	N/A	N/A	N/A	N/A	N/A
Peonidin-3-O-arabinoside	0.0463698347	0.044280496	0.0426115675	N/A	N/A	N/A	0.01573594	0.01470546	0.01730138
Peonidin-3-(caffeoyl-glucosyl-glucoside)-5-glucoside	N/A	N/A	N/A	N/A	N/A	N/A	N/A	N/A	N/A
Peonidin-3-sophoroside-5-glucoside	N/A	N/A	N/A	N/A	N/A	N/A	N/A	N/A	N/A
Peonidin-3-O-5-O-(6-O-coumaroyl)-diglucoside	N/A	N/A	N/A	N/A	N/A	N/A	N/A	N/A	N/A
Peonidin-3,5-O-diglucoside	12.9576446	12.312366	11.9262241	3.27135005	3.51530956	3.18446317	4.16726	4.03852	4.4433

Peonidin-3-O-(6-O-malonyl-beta-D-glucoside)	0.697134986	0.613575737	0.640246467	N/A	N/A	N/A	0.193178	0.1762696	0.1951608
Peonidin-3-O-glucoside	22.2930441	21.1781166	20.6695366	0.0161930569	0.0197480436	0.012698026	7.04866	6.98522	7.10364
Peonidin-3-O-rutinoside	742.940771	733.106568	716.023661	N/A	N/A	N/A	276.914	276.194	271.598
Peonidin-3-O-caffeoyl-feruloyl-sophoroside-5-glucoside	N/A	N/A	N/A	N/A	N/A	N/A	N/A	N/A	N/A
Peonidin-3-O-(6-O-p-coumaroyl)-glucoside	14.8479339	13.2036528	13.962537	N/A	N/A	N/A	4.10212	3.95878	4.0869
Petunidin-3-O-galactoside	17.9415978	17.2968834	17.091883	N/A	N/A	N/A	7.30028	7.33514	7.391
Petunidin-3-O-(6-O-malonyl-beta-D-glucoside)	N/A	N/A	N/A	N/A	N/A	N/A	N/A	N/A	N/A
Petunidin-3-O-(6-O-p-coumaroyl)-glucoside	N/A	N/A	N/A	N/A	N/A	N/A	N/A	N/A	N/A
Petunidin-3-O-5-O-(6-O-coumaroyl)-diglucoside	N/A	N/A	N/A	N/A	N/A	N/A	N/A	N/A	N/A
Petunidin-3,5-O-diglucoside	N/A	N/A	N/A	N/A	N/A	N/A	N/A	N/A	N/A
Petunidin-3-O-glucoside	3.83285124	3.54718499	3.44880053	N/A	N/A	N/A	1.490794	1.46163	1.465778
Petunidin-3-O-rutinoside	N/A	N/A	N/A	N/A	N/A	N/A	N/A	N/A	N/A
Petunidin-3-O-arabinoside	N/A	N/A	N/A	N/A	N/A	N/A	N/A	N/A	N/A
Petunidin-3-O-sambubioside-5-O-glucoside	N/A	N/A	N/A	N/A	N/A	N/A	N/A	N/A	N/A
Petunidin-3-O-sophoroside	N/A	N/A	N/A	N/A	N/A	N/A	N/A	N/A	N/A
Petunidin-3-O-sambubioside	N/A	N/A	N/A	N/A	N/A	N/A	N/A	N/A	N/A
Procyanidin A1	N/A	N/A	N/A	N/A	N/A	N/A	N/A	N/A	N/A
Procyanidin C1	N/A	N/A	N/A	N/A	N/A	N/A	N/A	N/A	N/A
Procyanidin B2	7.00323691	6.55465818	5.93296089	0.412455159	0.52337791	0.432834377	2.15686	2.15752	2.1697
Procyanidin A2	N/A	N/A	N/A	N/A	N/A	N/A	N/A	N/A	N/A
Procyanidin B1	0.242774105	0.21405496	0.205907986	0.0129779653	0.0136308569	0.0133189697	0.070074	0.0625666	0.0638172
Procyanidin B3	0.482193526	0.418374665	0.392323365	N/A	N/A	N/A	0.1193534	0.119143	0.1171456
Quercetin-3-O-glucoside	380.519972	353.153485	356.23398	37.0857763	40.7168895	38.0612422	124.8536	128.1436	120.2656
Naringenin-7-O-glucoside	2.92092975	2.47134718	2.48689451	4.70920444	5.20123824	5.10751083	1.917552	1.96913	2.0575
Kaempferol-3-O-rutinoside	7.57300275	7.1238941	6.35392705	14.0825458	14.8829123	15.0231584	4.85352	5.06272	3.95564
Rutin	694.989669	696.193029	640.729543	45.350675	50.332838	45.715118	226.258	222.38	193.0028

Dihydromyricetin	3.64321625	3.21848525	3.00802498	8.28466731	9.08766716	8.84997593	2.37136	2.2158	2.34328
Naringenin	0.231950069	0.234326408	0.207505751	0.151839923	0.125270431	0.114402022	0.0804574	0.0822724	0.1039818
Dihydrokaempferol	1.62213499	1.484437	1.45936904	18.6929605	19.9648341	19.5034665	4.65926	4.6226	4.6365
Afzelin	1.65068871	1.61187668	1.69703253	3.10564127	3.31300149	2.90183919	1.132984	1.119402	1.127362
Chalcone	N/A	N/A	N/A	N/A	N/A	N/A	N/A	N/A	N/A
